# Improvement in the signs and symptoms of dry eye disease with dobesilate eye drops

**DOI:** 10.1186/s40779-015-0068-8

**Published:** 2015-12-21

**Authors:** Pedro Cuevas, Luis Antonio Outeiriño, Carlos Azanza, Javier Angulo, Guillermo Giménez-Gallego

**Affiliations:** Facultad de Medicina, Universidad Alfonso X, Madrid, Spain; Departamento de Oftalmología, Hospital de Día Pío XII, Madrid, Spain; Servicio de Histología. Departamento de Investigación, IRYCIS; Hospital Universitario Ramón y Cajal, Madrid, Spain; Departamento de Estructura y Función de Proteínas, Centro de Investigaciones Biológicas. CSIC, Madrid, Spain

**Keywords:** Dry eye disease, Dobesilate eye drops, Fibroblast growth factor, Vascular endothelial growth factor

## Abstract

**Background:**

Dry eye is a multifactor disease of the tear film and ocular surface that substantially affects quality of life.

**Case presentation:**

Dobesilate administered as eye drops was well tolerated and effective in treating both the objective signs and subjective symptoms of dry eye disease in this 2-week study.

**Conclusion:**

To the best of our knowledge, this is the first clinical report of using dobesilate in eye drops. Dobesilate may provide a novel approach to treating drying diseases of the eye.

## Background

Dry eye disease (DED) is a progressive and multifactorial disease affecting the tear film and ocular surface that causes discomfort, visual disturbances and tear film instability with potential damage to the ocular surface [1]. DED is one of the most common ophthalmic problems, and it is estimated that up to one-third of the global population may be affected. Although the pathogenesis of DED is not fully understood, it is recognized that inflammation has a prominent role in the development and amplification of the signs and symptoms of DED [2–5]. Accordingly, successful application of anti-inflammatory medications in the treatment of DED provides hope for the millions of individuals who suffer from this deleterious condition. Herein, we report the safety and effectiveness of topical administration of the anti-inflammatory drug dobesilate [6–9] in patients with severe DED.

### Patients and treatment

Eight patients (seven women and one man) with severe DED in both eyes participated in this study. The study was approved by our institutional ethical committee, and patients signed an informed consent form that included a comprehensive description of the proposed procedure with dobesilate. Patients were asked before and during treatment to describe the DED symptoms they experienced. DED symptoms (foreign body sensation, dryness, photophobia, eye pain, and blurred vision) were scored from 0 to 4, with a score of 0 indicating no symptoms and a score of 4 indicating very severe symptoms. DED signs were assessed by Schirmer’s tear test (STT) and by fluorescence corneal staining (FCS). STT performed without anesthesia measures tear volume. For FCS, 5 μl of 0.5 % fluorescein solution was instilled in the conjunctival sac. Fluorescein diffuses rapidly into the corneal stroma when there is a loss of epithelial integrity. Corneal staining was examined under standard illumination using a slit-lamp microscope with a cobalt filter. All patients initially had STT scores <5 mm/5 min and corneal epitheliopathy in both eyes. DED symptom scores and signs were compared at baseline, the second week of treatment, and 4 weeks after treatment discontinuation.

Furthermore, patient satisfaction scores were assessed during treatment using four questions adapted from the Study Group on Impact of Dry Eye on Everyday Life (IDEEL) questionnaire [10], with minor modifications. The four questions included the following: 1) my eyes feel dry in the morning, 2) my eyes feel dry at the end of the day, 3) my eyes feel refreshed when I use dobesilate, and 4) I frequently forget my symptoms when I use dobesilate.

Patients consented to treatment with dobesilate eye drops, which were prepared in the pharmacy service of our institution. Patients self-administered dobesilate eye drops (1 drop/eye/twice a day) for 2 weeks. Dobesilate was used as a 12.5 % solution of diethylammonium 2-5-dihydroxybenzene sulfonate (etamsylate, Dicynone®, Sanofi-Aventis, Paris, France). Quantitative comparisons between baseline and post-treatment symptom scores were performed for 16 eyes (8 patients) using paired t-tests. A p value less than 0.05 was considered statistically significant.

**Fig. 1:**
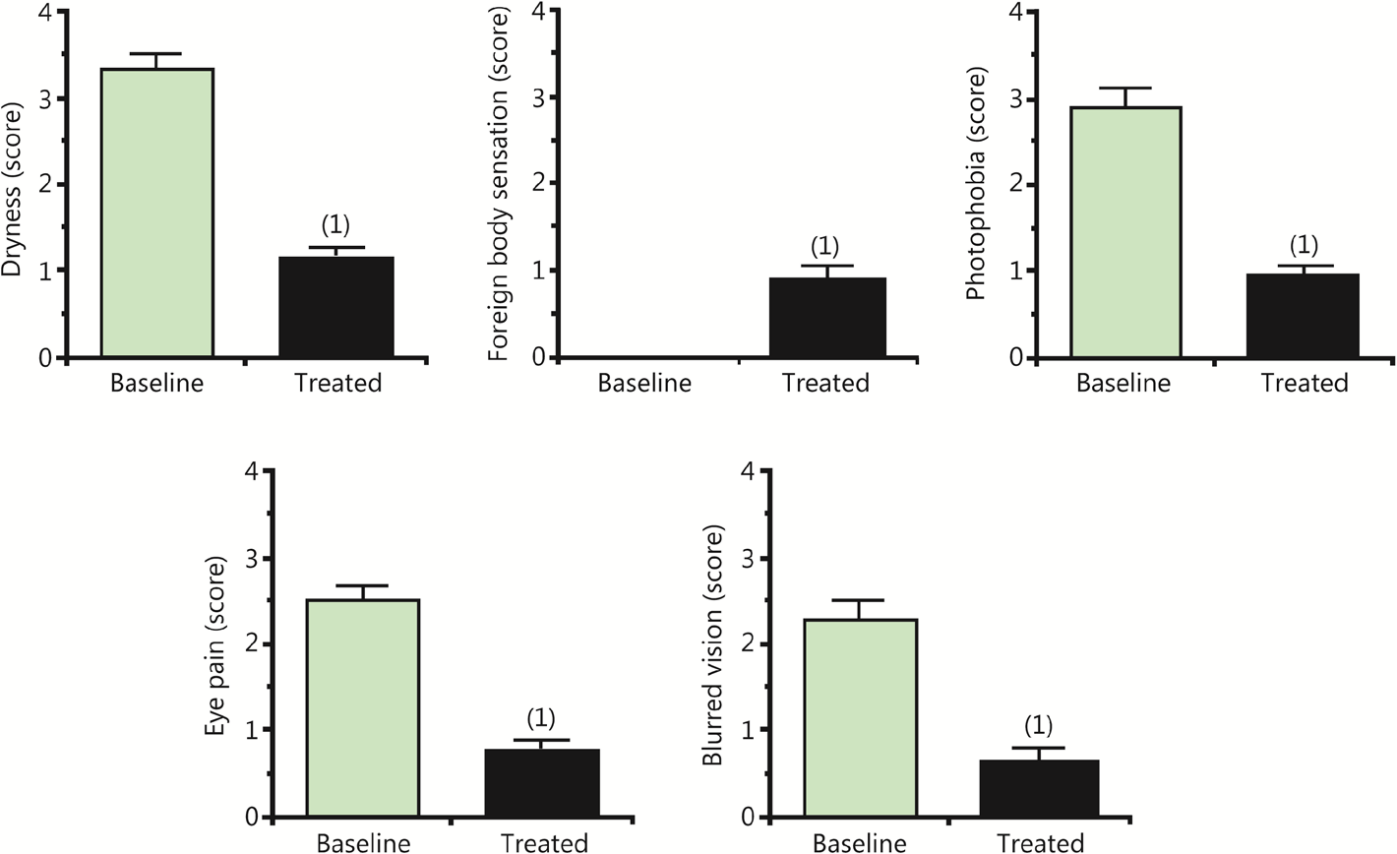
Improvement of dry eye symptoms after dobesilate eye drop instillation for two weeks. Data from 16 eyes (8 patients) are expressed as the mean ± SEM *** indicate *p* < 0.001 vs. baseline by paired *t*-test

Symptoms of DED were significantly improved in all patients after treatment (Fig. 1). In addition, STT and corneal epitheliopathy assessed by FCS studies improved significantly. Furthermore, patients exhibited good compliance and did not report any adverse effects related to dobesilate treatment. As an example of the effectiveness of dobesilate eye drops, we present a patient with severe DED who participated in the present study.

## Case presentation

**Fig. 2:**
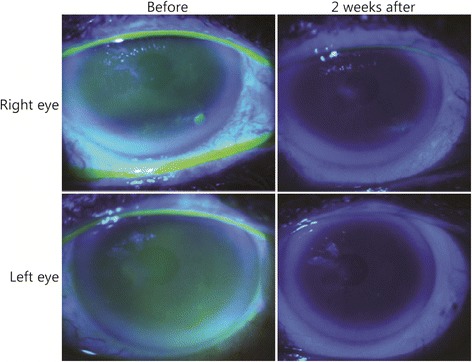
Slit-lamp fluorescein corneal staining pictures showed that inferior corneal punctuate epitheliopathy disappeared within 2 weeks of the instillation of dobesilate eye drops. The corneal erosion (*) improved 2 weeks after administration of the drug

This is a representative case selected from among the eight patients with DED who were treated with dobesilate eye drops. A 68-year-old Caucasian woman with a five-year clinical history of DED in both eyes presented with obvious ocular symptoms. The patient had been previously treated with artificial tears, but without success. STT scores were <5 mm/5 min in both eyes. Punctuate epithelial erosions on the inferior corneal surface were present in both eyes (Fig. 2). These punctuate corneal epitheliopathies were most noticeable in the right eye (Fig. 2). Furthermore, the right eye showed a conspicuous corneal erosion (Fig. 2). These findings indicated a severe DED.

**Fig. 3:**
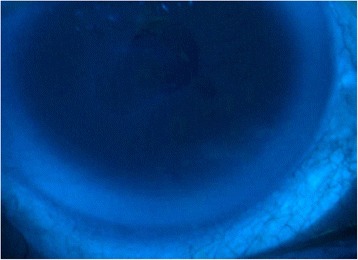
Slit-lamp fluorescein corneal staining image of the right eye showing complete resolution of corneal ulceration 4 weeks after treatment discontinuation

After 2 weeks of dobesilate treatment, STT scores improved in both eyes to 12 mm/5 min in the right eye and 15 mm/5 min in the left eye. Corneal epitheliopathy improved as well, and corneal healing was practically achieved at the same time (Fig. 2). In addition, the patient’s subjective symptoms improved significantly after 2 weeks of treatment. At 4 weeks after treatment discontinuation, the STT score was 15 mm/5 min in the right eye and 20 mm/5 min in the left eye, and the corneal ulceration of the right eye was healed (Fig. 3). The patient exhibited good compliance to dobesilate treatment; the data showed optimal tolerability, a lack of adverse effects upon instillation and high patient satisfaction. In particular, the patient did not report any adverse events of blurring, itching or scratching upon instillation. Instead, the patient reported a statistically significant reduction in all subjective symptoms. Objective and subjective data were recorded at 4 weeks after treatment discontinuation, and the patient reporting using artificial tears only sporadically.

## Discussion and conclusion

Dry eye is defined by the International Dry Eye Workshop as a multifactor disease of the tear film and ocular surface [1] that substantially affects quality of life because of symptoms that include pain and irritation. DED has a negative effect on ocular health and the patient’s general health and well-being, as DED often disrupts daily activities [11, 12]. This condition is more prevalent in women and increases with age. The prevalence of DED in the population has been reported to be as high as 34 % [13, 14], constituting a public health problem and an economic burden [15]. Risk factors for the development of DED include advanced age, female sex, hormonal imbalance, autoimmune disease, vitamin deficiency, abnormal corneal innervation, environmental stress, contact lens use, medication, computer use, and ophthalmic surgery [8].

The ocular surface (cornea, conjunctiva and accessory lachrymal glands), meibomian glands, main lachrymal gland and interconnecting neural reflex loops constitute a functional unit [16]. In DED, inflammation affects all of the components in the functional unit, which suggests that immunological circuits are an integrate part of the system [2, 17, 18]. Its central core is characterized by cyclic events that interconnect tear film instability, tear hyperosmolarity, local inflammatory responses and metaplastic changes in the ocular surface epithelia. The purpose of any therapeutic approach is to interrupt this type of cycle at any point to slow or prevent the disease progression [8].

The recommended treatments for mild DED are lifestyle changes and use of artificial tears [17]; however, frequent instillation is often required. Furthermore, patients with moderate to severe disease may require anti-inflammatory medications or surgery [17]. Cyclosporine ophthalmic emulsion has been approved by the United States Food and Drug Administration for treating moderate to severe DED. Clinical data indicate that long-term treatment with cyclosporine A 0.05 % ophthalmic emulsion can yield positive results with regard to objective and subjective findings, including corneal surface staining, STT scores, blurred vision, and frequency of artificial tear application [19]. However, some patients experience bothersome adverse effects (e.g., pain, burning or irritation) that impact medication tolerability. Topical corticosteroid treatment has demonstrated efficacy in clinical trials at diminishing symptom severity and minimizing ocular surface staining in patients with DED [20, 21]. Systemic corticosteroid administration may also be effective in the management of DED [22]. Unfortunately, long-term topical or systemic corticosteroid use is associated with deleterious adverse effects, such as ocular hypertension, risk of cataract formation and opportunistic infections. Orally administered anti-inflammatory tetracycline derivatives have been used to treat DED secondary to ocular rosacea and blepharitis [23]. However, despite extensive evidence from experimental trials indicating their potential benefits in treating DED, there is limited clinical evidence of their efficacy.

Because existing DED treatments have serious concerns, the search for a safe and efficient therapy for DED is urgently needed. Inflammation of the ocular surface in DED is sustained by ongoing activation and infiltration of pathogenic immune cells, primarily of the CD4+ T cell compartment [24]. These biological processes were favored and maintained by lymphangiogenesis [25]. Interestingly, targeting prolymphangiogenic growth factors, such as vascular endothelial growth factor (VEGF), or their receptors improved murine DED, which is reflected by decreased inflammation [26]. It was reported that fibroblast growth factor (FGF) simultaneously provokes hemangiogenesis and lymphangiogenesis on the cornea through differential expression of VEGF [27]. Thus, inhibition of FGF-driving inflammatory lymphangiogenesis is a potential therapeutic target for DED.

Dobesilate, a drug with a long history of clinical safety [28], has been used for many years as a vasculotropic drug. Recently, it was reported that dobesilate is a powerful inhibitor of FGF [29], which is a potent pro-inflammatory protein [30, 31]. FGF is a strict mediator of VEGF activities [29, 32, 33], and some of these activities were also abolished by dobesilate [34]. Dobesilate shows a marked anti-inflammatory activity in several inflammation-dependent diseases and inflammation models [6–9]. Thus, it seems obvious that these activities may, at least in part, contribute to an improvement in the signs and symptoms of DED, which was observed with dobesilate treatment in the present report.

The results of our study are very encouraging because of the magnitude and consistency of the patients’ responses to the treatment and the rarity of spontaneous improvements in patients with DED. Although further large-scale therapeutic trials are necessary to definitively establish the efficacy of this treatment, the results presented in this report seem to provide a basis for undertaking these trials.

## Consent

Written informed consent for publication of the clinical details and images was obtained.

## Abbreviations

DED: dry eye disease
FCS: fluorescence corneal staining
FGF: fibroblast growth factor
IDEEL: impact of dry eye on everyday life
STT: Schirmer’s tear test
VEGF: vascular endothelial growth factor
